# Type II restriction modification system in *Ureaplasma parvum* OMC-P162 strain

**DOI:** 10.1371/journal.pone.0205328

**Published:** 2018-10-16

**Authors:** Heng Ning Wu, Yukiko Nakura, Michinobu Yoshimura, Ourlad Alzeus Gaddi Tantengco, Makoto Nomiyama, Toshimitsu Takayanagi, Tomio Fujita, Kiyoshi Yasukawa, Itaru Yanagihara

**Affiliations:** 1 Department of Developmental Medicine, Research Institute, Osaka Women’s and Children’s Hospital, Izumi City, Osaka, Japan; 2 College of Medicine, University of the Philippines Manila, Manila, Philippines; 3 Department of Obstetrics and Gynecology, National Hospital Organization, Saga Hospital, Saga, Japan; 4 Department of Pediatrics, National Hospital Organization, Saga Hospital, Saga, Japan; 5 Fujita Clinic, Osaka City, Osaka, Japan; 6 Division of Food Science and Biotechnology, Graduate School of Agriculture, Kyoto University, Sakyo-ku, Kyoto, Japan; Academia Sinica, TAIWAN

## Abstract

*Ureaplasma parvum* serovar 3 strain, OMC-P162, was isolated from the human placenta of a preterm delivery at 26 weeks’ gestation. In this study, we sequenced the complete genome of OMC-P162 and compared it with other serovar 3 strains isolated from patients with different clinical conditions. Ten unique genes in OMC-P162, five of which encoded for hypothetical proteins, were identified. Of these, genes *UPV_*229 and *UPV_*230 formed an operon whose open reading frames were predicted to code for a DNA methyltransferase and a hypothetical protein, respectively. DNA modification analysis of the OMC-P162 genome identified N4-methylcytosine (m4C) and N6-methyladenine (m6A), but not 5-methylocytosine (m5C). UPV230 recombinant protein displayed endonuclease activity and recognized the CATG sequence, resulting in a blunt cut between A and T. This restriction enzyme activity was identical to that of the cultivated OMC-P162 strain, suggesting that this restriction enzyme was naturally expressed in OMC-P162. We designated this enzyme as *Upa*P162. Treatment of pT7Blue plasmid with recombinant protein UPV229 completely blocked *Upa*P162 restriction enzyme activity. These results suggest that the *UPV_*229 and *UPV_*230 genes act as a type II restriction-modification system in *Ureaplasma* OMC-P162.

## Introduction

*Ureaplasma* spp. belong to the class *Mollicutes* [1] and have been implicated in the morbidity and mortality of newborns [2–4]. *Ureaplasma* spp. range from 100 nm to 1 μm in size [5] and are among the smallest self-replicating organisms lacking a peptidoglycan layer [1]. Historically, these human pathogens have been divided into 14 serovars that can be classified into two major groups: *U*. *parvum* (serovars 1, 3, 6, and 14) and *U*. *urealyticum* (serovars 2, 4, 5, and 7–13) [6, 7]. Unlike *Mycoplasma* spp., *Ureaplasma* spp. hydrolyze urea via urease to generate 95% of their ATP [8, 9].

*Ureaplasma* spp. commonly colonize the human urogenital tract [10]. Their genome is 0.72–0.95 megabase pairs and contains 571–711 predicted coding DNA sequences (CDSs), of which 30% have been deduced as hypothetical proteins [11, 12]. Their genome contains five putative virulence factors: urease [13], multiple-banded antigen (MBA) [14], hemolysin [15], serine/threonine kinase, and protein phosphatase [16–18]. MBA has been extensively studied. It is a major surface-exposed lipoprotein that activates the nuclear factor-kappa B pathway via TLR2 and initiates inflammation via the N-terminal diacylated lipopeptide. This results in preterm delivery and intrauterine fetal death in mice [14].

Bacteria can incorporate extracellular DNA into their genome, which may be accompanied by a characterization or virulence change. *U*. *parvum* serovar 3 type strain ATCC700970 was isolated from a patient with nongonococcal urethritis [15], and the clinical strain SV3F4 of *U*. *parvum* serovar 3 was isolated from a Japanese patient with history of an infectious abortion [12]. In our previous study, the complete genome of the SV3F4 clinical strain was sequenced, and its genome was found to be the smallest among all 14 other human pathogenic serovars of *Ureaplasma* spp. [12]. This genome size variation might be caused by gene insertions and deletions (indels). A good understanding of the genetic variations among bacterial strains could contribute toward developing future strategies for the diagnosis and treatment of bacterial infections. However, this has not been investigated in *Ureaplasma* spp. In our present study, we determined the whole genome sequence of OMC-P162 strain and identified a novel type II restriction modification system in OMC-P162.

## Materials and methods

### Bacterial strains and cultures

All of the *U*. *parvum* clinical strains used in this study and ATCC700970 were cultured at 37°C in *Ureaplasma* medium [2], consisting of 2.4% *Mycoplasma* broth base, 2.5% yeast extract, 5% horse serum, 0.04% urea, 0.01% L-cysteine hydrochloride, 0.001% phenol red, and 1,000 U/ml penicillin. The *Escherichia coli* JM109 (Takara Bio, Shiga, Japan) and C43 (DE3) (Lucigen, WI, USA) strains were used for DNA manipulation and recombinant protein expression, respectively. These two strains were grown at 37°C in Luria–Bertani (LB) medium. This study was conducted in accordance with the Declaration of Helsinki and was approved by the Osaka Medical Center for Maternal and Child Health (former name of the Osaka Women's and Children's Hospital) Ethics Committee. Written informed consent was obtained from individuals or legally acceptable representatives when the patients were infants.

### DNA extraction, plasmid construction and PCR mutagenesis

*Ureaplasma* genomic DNA was prepared by Genomic DNA Buffer Set (Qiagen, Hilden, Germany) and isolated by QIAGEN Genomic-tip 20/G (Qiagen, Hilden, Germany), which was then amplified by polymerase chain reaction (PCR) using Ex Taq (Takara Bio, Shiga, Japan) for plasmid construction. To prepare the PCR product for the pUPV-230 plasmid construction, the primer pair UP162-F15 (5ʹ-ctagctagcatgatggacaattatgttag-3ʹ) and UP162-R15 (5ʹ-ccgctcgagatggcgtccattataaatatc-3ʹ) was used. The PCR protocol consisted of 2 min at 94°C; 30 cycles of 15 s at 94°C, 15 s at 45°C, and 1 min at 72°C; followed by 1 min at 72°C.

To prepare a PCR product with a mutated TGA codon (Trp in *Mycoplasma*) to TGG (Trp) for pUPV-229 plasmid construction, overlap extension PCR was performed over two rounds of PCR. We designed three sets of primers. The outermost primers were methyl-F1 (5ʹ-gggcatatgaaaatatcgtacaataa-3ʹ) and methyl-R1 (5ʹ-ccgctcgagacataattgtccatcatttt-3ʹ), and the two sets of inner primers were methyl-F2 (5ʹ-atataatgagtgggttaaatc-3ʹ) and methyl-R2 (5ʹ-gatttaacccactcattatat-3ʹ), and methyl-F3 (5ʹ-ctgtagggtgggcattagaa-3ʹ) and methyl-R3 (5ʹ-ttctaatgcccaccctacag-3ʹ). Both sets of inner primers were sense and antisense oligonucleotides where the TGA codon was changed to TGG. During the first round of PCR, the products were amplified using the methyl-F1 and methyl-R2, methyl-F2 and methyl-R3, and methyl-F3 and methyl-R1 primers. The PCR protocol consisted of 2 min at 94°C; 30 cycles of 15 s at 94°C, 15 s at 45°C, and 1 min at 72°C; followed by 1 min at 72°C. These three PCR products were purified and then mixed. The mixture was used as template in the second round of PCR using the outermost primers methy-F1 and methy-R1. The PCR protocol consisted of 2 min at 94°C; 30 cycles of 15 s at 94°C, 15 s at 50°C, and 1 min at 72°C; followed by 1 min at 72°C. Both pUPV-229 and pUPV-230 plasmids were generated by using the *Nde*I and *Xho*I sites in the *E*. *coli* expression plasmid pET28a(+) (Novagen, MA, USA). To prepare substrates for UPV229 and UPV230, PCR products with a mutated CATG were constructed by three sets of specifically designed primers. The outermost primers were pT-F1 and pT-R3, and the two sets of inner primers were pT-F3 and pT-R7, and pT-F7 and pT-R2. Both sets of inner primers were sense and antisense oligonucleotides where the CATG motif had been mutated. During the first round of PCR, the products were amplified using pT-F1 and pT-R7, pT-F3 and pT-R2 and pT-F2 and pT-R3. PCR was performed as described above and generated product 8. pT-F1 and pT-R1 were used to amplify product 1, pT-F2 and pT-R2 for product 2, pT-F3 and pT-R3 for product 3, pT-F4 and pT-R4 for product 4, pT-F5 and pT-R5 for product 5, pT-F6 and pT-R6 for product 6, and pT-F4 and pT-R5 for product 7. S1 Table lists the sequences of the primers used.

### Protein analysis

The proteins were analyzed by sodium dodecyl sulfate-polyacrylamide gel electrophoresis (SDS-PAGE) and western blotting. Following the 10% SDS-PAGE, the samples were stained with Coomassie brilliant blue. For western blotting, the proteins in the SDS-PAGE gel were transferred onto a nylon sheet (GE Healthcare, Freiburg, Germany) and tagged with anti-His antibody (Cell Signaling Technology, MA, USA). Following treatment with horseradish peroxidase-conjugated secondary antibody (Rockland Immunochemicals, PA, USA), the tagged proteins were visualized via chemiluminescence (LAS-4000; Fujifilm, Tokyo, Japan). The function of proteins was predicted by InterPro analysis (http://www.ebi.ac.uk/interpro/index.html).

### Next-generation sequencing, genome assembly, annotation, DNA modification analysis, and genome comparison

Genomic DNA from OMC-P162 was extracted and purified using a Qiagen genomic DNA kit (Qiagen, Hilden, Germany) according to the manufacturer’s instructions. The insertion library of *U*. *parvum* was prepared and sequenced using a PacBio RS II single-molecule real-time sequencing (SMRT) system (Pacific Biosciences, CA, USA). We obtained 85,709 filtered reads from one SMRT Cell, with an average read length of 10,316 base pairs (bp). These were assembled into one contig of 732,031 bp using the Hierarchical Genome Assembly Process (HGAP) 2.0 (Pacific Biosciences) [19]. This contig was trimmed and circularized into a final complete genome of *U*. *parvum* OMC-P162 strain. A diagram of the overall genomic structure was generated using DNAPlotter 1.10 from Artemis 15.0 (Wellcome Sanger Institute, https://www.sanger.ac.uk/science/tools/artemis) [20]. The predicted CDSs, 6 rRNA genes, and 30 tRNA genes were annotated by a BLAST-based search (https://blast.ncbi.nlm.nih.gov/Blast.cgi) against the GenBank nucleotide and non-redundant databases. DNA modification analysis was carried out using RS Modification Detection 1 in SMRT Analysis v2.3.0 software and visualized with SMRT View software (PacBio,CA, USA; https://www.pacb.com/products-and-services/analytical-software/smrt-analysis/). Interpulse durations (IPDs) were measured as described previously [21]. The genome comparison was performed using wgVISTA (http://genome.lbl.gov/cgi-bin/WGVistaInput). The prediction of promoters was searched on the website of BDGP (http://www.fruitfly.org/seq_tools/promoter.html).

The genome sequence for the *U*. *parvum* strain OMC-P162 was deposited in the GenBank database (accession no. AP018561).

### *Upa*P162 expression and purification

To purify the *Upa*P162 enzyme from OMC-P162, cells from a 1,000 ml culture were centrifuged at 26,000 × *g* for 60 min at 4°C and gently suspended in 10 ml of ice-cold phosphate-buffered saline (PBS) consisting of 75 mM Na_3_PO_4_ (pH 7.3) and 68 mM NaCl. The cells were recovered by centrifugation at 26,000 × *g* for 60 min at 4°C, suspended in 1 ml PBS containing 1% Triton X-100 and 0.1 mM phenylmethylsulfonyl fluoride (PMSF) (Sigma-Aldrich, Steinheim, Germany), and incubated for 60 min on ice. The cell lysate was centrifuged at 26,000 × *g* for 60 min at 4°C. Then, the supernatant was diluted 10-fold with buffer A (50 mM Tris-HCl, pH 7.5) and loaded on a Macro-Prep High S Support column (Bio-Rad, CA, USA) equilibrated with 1 ml of buffer A, washed with 2 ml of buffer A, and eluted with 1 ml of buffer A at various NaCl concentrations. The restriction enzyme-rich fraction was diluted with buffer A to a final concentration of 100 mM NaCl, and 1 ml was loaded on an equilibrated Heparin Sepharose 6 Fast Flow column (GE Healthcare, Uppsala, Sweden) filled with buffer B (10 mM Tris-HCl [pH 7.5], 0.1 mM EDTA, 10% glycerol, 1 mM dithiothreitol [DTT], and 100 mM NaCl). The column was washed with 2 ml of buffer B, and the fraction was eluted with 1 ml of buffer B at various NaCl concentrations. The restriction enzyme-rich fraction was recovered and dialyzed in buffer B and stored at −80°C.

To induce the expression of the UPV230 protein, the *E*. *coli* strain C43 (DE3) harboring pUPV-230 was grown in 1,000 ml of culture medium at 37°C until the optical density at 580 nm (OD_580_) reached 0.5. Then, the expression was induced with 0.4 mM of isopropyl-β-D-1-thiogalactopyranoside (IPTG) and allowed to proceed at 25°C overnight. The cells were pelleted by centrifugation at 6,000 × *g* for 30 min at 4°C and then resuspended in 50 ml of ice-cold buffer C (50 mM Tris-HCl [pH 7.5] and 300 mM NaCl) containing 1 mM PMSF, 10 mM imidazole, and 10% glycerol. This was followed by 10 cycles of sonication on ice (one cycle consisted of 60 s of sonication and 60 s of resting) with a sonicator UD-200 (TOMY seiko, Tokyo, Japan) equipped with a microprobe at an output of 6. The suspension was centrifuged at 34,000 × *g* for 60 min at 4°C, and the supernatant was passed through a 0.22 μm syringe filter. The filtered supernatant was loaded in a 6 ml equilibrated column of cOmplete His-Tag Purification Resin (Roche, Mannheim, Germany) with buffer C. The column was washed with 60 ml of buffer C containing 10 mM imidazole. Then, the protein was eluted with 6 ml of buffer C containing 500 mM imidazole. The eluted proteins were diluted with buffer A to a final concentration of 100 mM NaCl and loaded on 3 ml equilibrated Macro-Prep High S Support column by buffer D (50 mM Tris-HCl [pH 7.5] and 100 mM NaCl). The column was washed with 6 ml of buffer D and eluted with 3 ml of buffer D containing various concentrations of NaCl. The restriction enzyme-rich fraction was further purified using a sepharose–heparin-filled column as described above.

The purification of the UPV229 recombinant protein was performed as described for the purification of the UPV230 recombinant protein with some modifications. Briefly, the *E*. *coli* C43 (DE3) strain harboring the pUPV-229 plasmid was grown in 400 ml of LB medium at 37°C until the OD_580_ reached 0.5. The expression was induced by IPTG, and the cells were recovered and disrupted. Then, the supernatant was collected and filtered. The UPV229 protein was purified using a cOmplete His-Tag Purification Resin-filled column followed by a sepharose–heparin-filled column.

### Determination of UPV230 restriction enzyme activity and its inhibitory activity of UPV229

To study the restriction enzyme activity in *Ureaplasma* cells, cultured OMC-P162 and C43 (DE3) strains in the mid-log growth phase were centrifuged at 17,800 × *g* for 20 min at 4°C, gently washed once with ice-cold PBS, suspended in PBS containing 1% Triton X-100, and incubated for 10 min on ice. Then, the restriction enzyme activity was assessed by incubating 1 μl of cell lysate and 1 μg of closed circular pT7Blue plasmid (Novagen, MA, USA) in a total volume of 20 μl containing buffer L (100 mM Tris-HCl [pH 7.5], 100 mM MgCl_2_, and 10 mM DTT; Takara Bio Inc, Shiga, Japan) for 1 h at 37°C. The reaction products were visualized by 1% agarose gel electrophoresis and ethidium bromide staining.

The endonuclease activity of the purified protein from the OMC-P162 and *UPV_*230 expressed in C43 (DE3) strains was assessed by incubating the purified protein and 1 μg of closed circular pT7Blue plasmid in a total volume of 20 μl containing L buffer at 37°C for the various times indicated. To analyze the endonuclease activity of UPV230 on PCR products and the *Ureaplasma* genome, the reaction pH was adjusted to 9.2. *Nla*III was purchased from New England Biolabs (Tokyo, Japan), and *Sph*I was purchased from Takara Bio (Otsu, Shiga, Japan). The reaction products were visualized by 1% or 3% agarose gel electrophoresis and ethidium bromide staining.

The inhibitory activity of UPV229 against the endonuclease activity of UPV230 was assessed by incubating purified UPV229 recombinant protein and 1 μg of closed circular pT7Blue plasmid or 250 ng of PCR products in a total volume of 20 μl containing methylation buffer (20 mM Tris-HCl [pH 7.5], 0.2 mM DTT, 0.5 mM EDTA, 5% glycerol, and 4 μM S-adenosylmethionine) at 37°C for 1 h. The plasmid or PCR products was recovered by phenol extraction and ethanol precipitation and digested with UPV230 protein. The DNA nucleotide sequence of the restriction site was determined using the BigDye Terminator v3.1 kit on an ABI 3130 Genetic Analyzer (Thermo Fisher Scientific, MA, USA).

## Results and discussion

### OMC-P162 genome, its modification, and genome comparison

**Fig 1:**
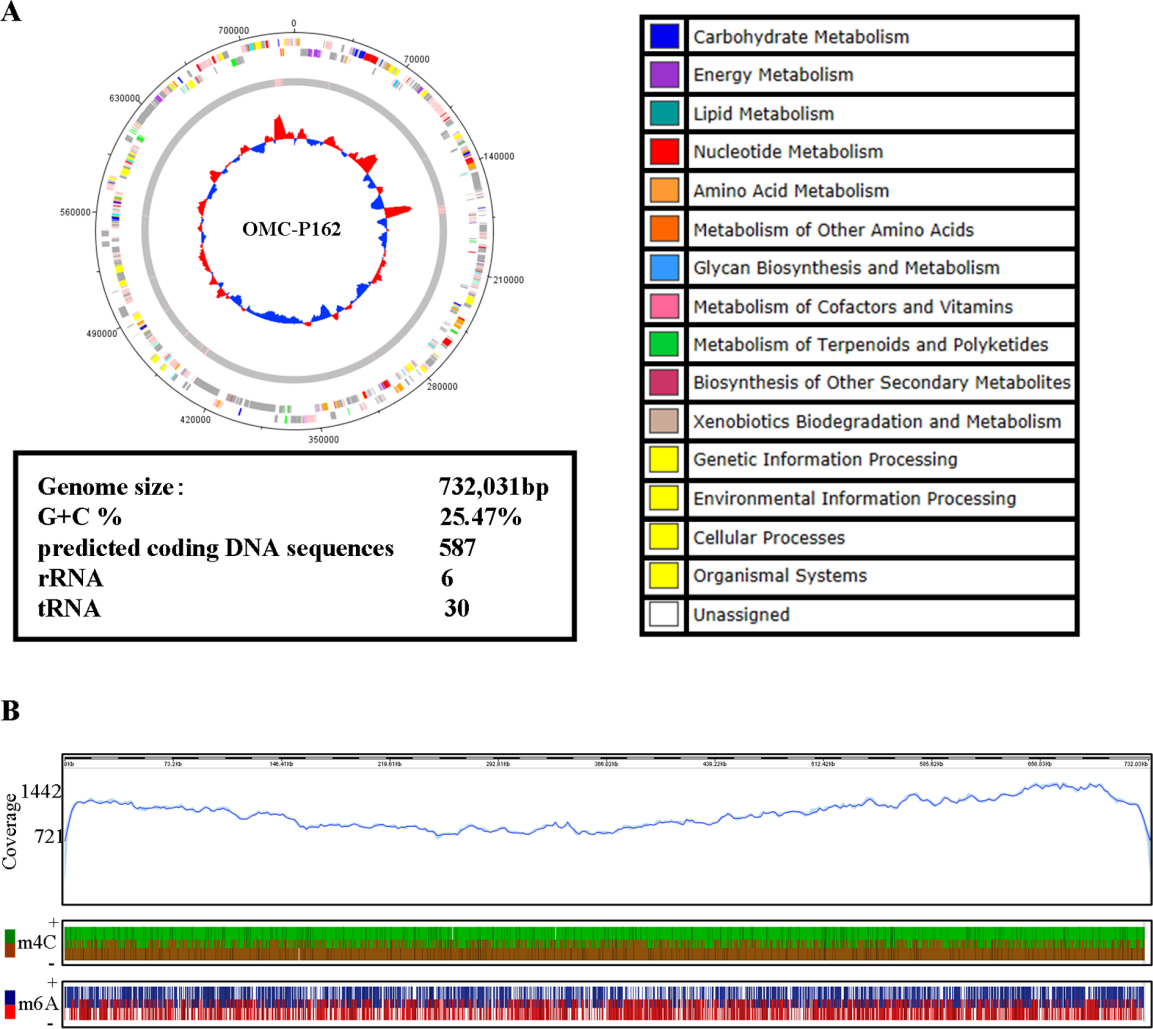
Complete genome sequence of *Ureaplasma* OMC-P162 strain and DNA modification. (A) The distribution of genes was depicted on the two outermost concentric circles. First concentric circle: predicted coding DNA sequences (CDSs) on the plus strand. Second concentric circle: predicted CDSs on the minus strand. The gray portion of the middle circle represents the nucleotides sequences while the pink portion indicated tRNA and rRNA. The innermost circle represents the GC skew. This figure was generated using DNAPlotter: Release 1.10 from Artemis Release 15.0.0, Sanger Institute. CDSs were classified by KEGG pathway categories and color codes for KEGG pathway categories were shown in the right. The genome size, GC content and numbers of CDS, rRNA and tRNA were also shown. (B) The upper panel shows the coverage of sequence reads and the bottom two panels show modification types and numbers of modification events within 2 kb of the genome of the plus (+) and minus strands (−).

**Fig 2:**
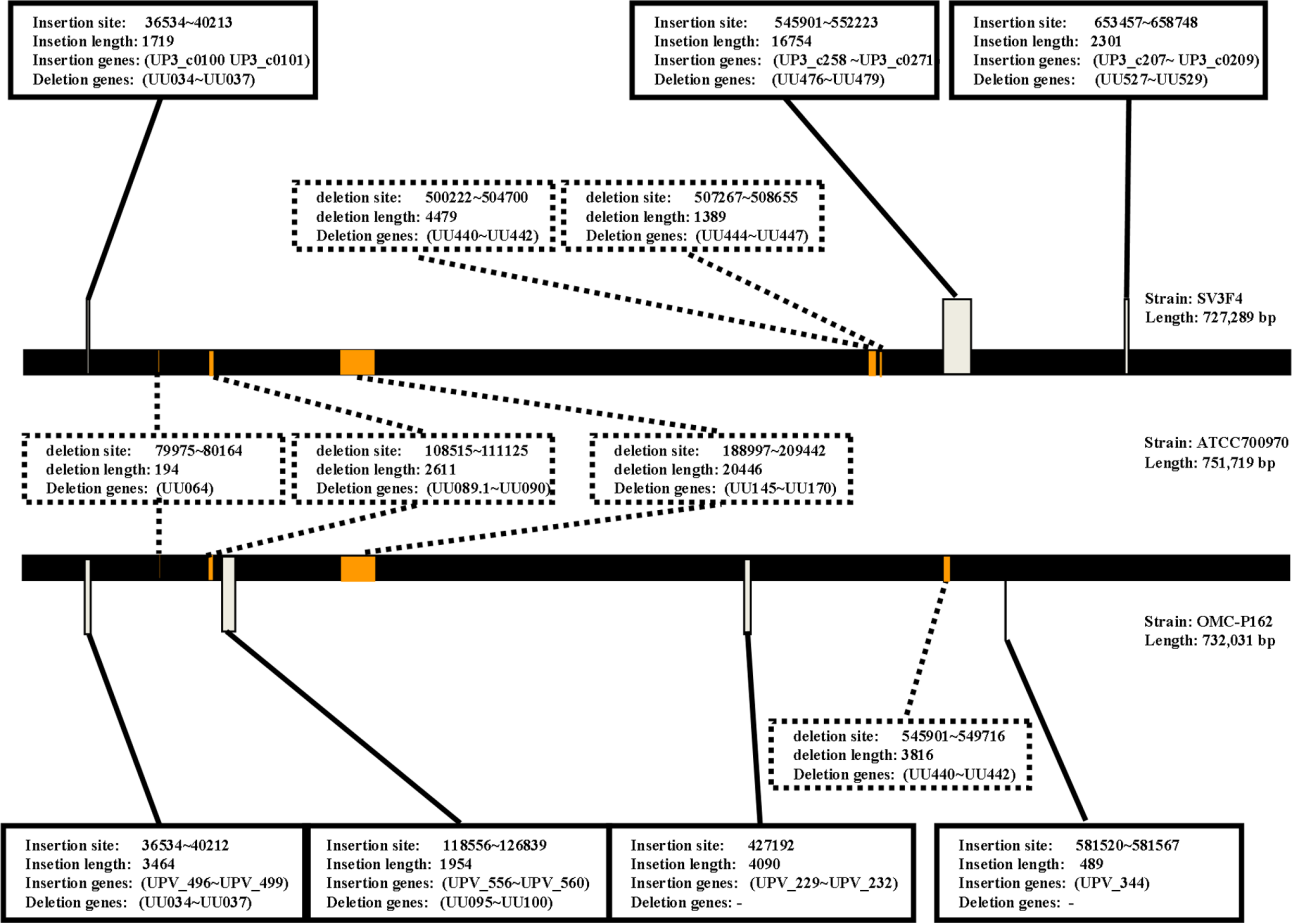
Comparison of the reference genome of ATCC700970 to the SV3F4 and OMC-P162 strains. ATCC700970 genome was shown as linear black bar. Upper panel shows genome comparison between ATCC700970 and SV3F4 strain, and the bottom panel shows genome comparison between ATCC700970 and OMC-P162 strain. Insertion and deletion genes were indicated as gray and orange bars, respectively. Insertion site, insertion length, insertion genes and deletion genes were shown in the square, while the deletion site, deletion length and deletion genes were shown in the dotted square.

**Fig 3:**
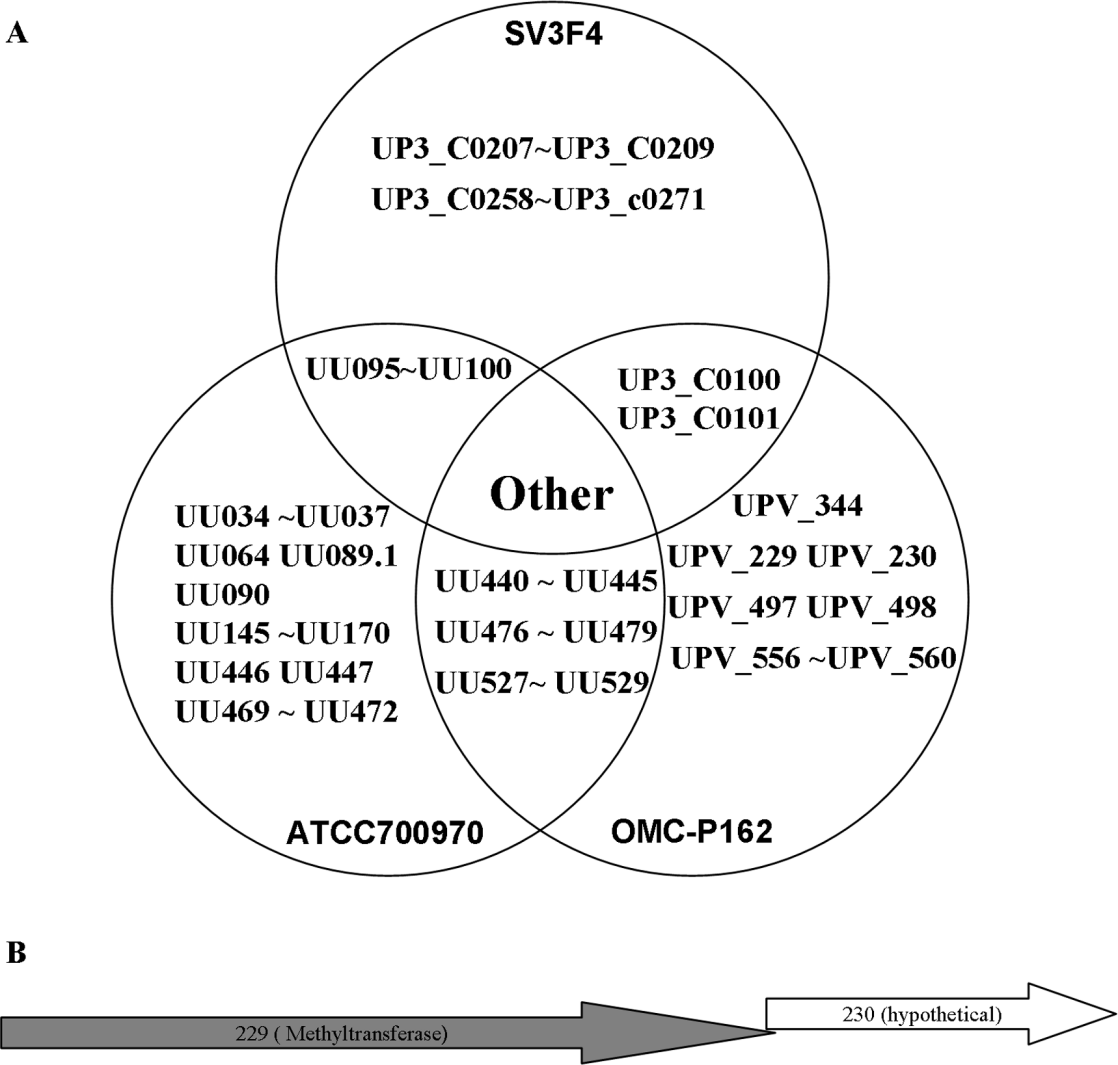
Comparison complete genome sequence of 3 strains. (A) Unique genes and shared genes among the strains SV3F4, ATCC700970 and OMC-P162 were shown by Venn diagram. (B) Organization of RM genes (not drawn to scale).

The length of the OMC-P162 genome was 732,031 bp with a G+C content of 25.47%. A total of 582 CDSs, 6 rRNA genes, and 30 tRNA genes were predicted (Fig 1A). The total number of post filtered subreads for DNA modification analysis was 920,879,592 bp. The estimated depth of coverage was 1,258, sufficient for m4C, m6A and m5C detection by IPD ratio. The recommended depths of coverage were 25, 25 and 250, respectively (https://www.pacb.com/wp-content/uploads/2015/09/WP_Detecting_DNA_Base_Modifications_Using_SMRT_Sequencing.pdf). The modified numbers of m6A nucleotides were 6,824, of m4C were 41,396 and of m5C were 0, while the numbers of unclassified modified nucleotides were estimated to be 225,981 (Fig 1B and S2, S3, S4 and S5 Tables). The comparison of the genomes of OMC-P162, ATCC700970, and SV3F4 identified four DNA fragments containing 14 gene insertions and seven accompanying gene deletions. Additionally, four DNA fragments containing 32 genes were deleted from the genome without gene insertion in the OMC-P162 genome. Three DNA fragments containing 19 genes were inserted into the genome and were accompanied by 11 gene deletions. Furthermore, five DNA fragments containing 36 genes were deleted from the genome without gene insertion in the SV3F4 genome (Fig 2 and S6 Table). Based on these data, we determined that the indel genes in the genome were clustered, and that 10 genes were unique to the OMC-P162 strain and were not present in ATCC700970 and SV3F4 strains (Fig 3A). Among these genes, *UPV_*229 and *UPV_*230 formed an operon structure. *UPV_*229 was predicted to code for a DNA (cytosine-5)-methyltransferase (Fig 3B) while *UPV_*230 might code for a restriction enzyme.

### Expression of restriction enzymes in *U*. *parvum* serovar 3

**Fig 4:**
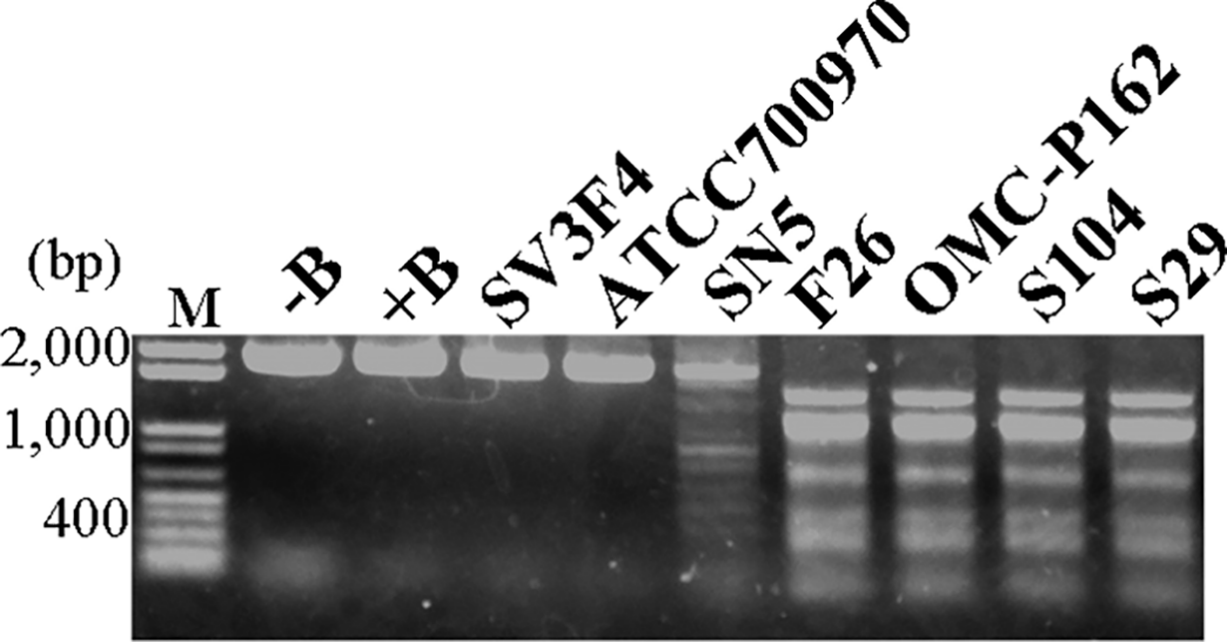
Detection of restriction enzyme in *Ureaplasma* spp. Comparison of restriction patterns of pT7Blue plasmid treated with whole-cell lysate of the indicated *Ureaplasma* spp. strains after incubation with L buffer for 1 h at 37°C. These products were separated by 1% agarose gel electrophoresis. pT7Blue plasmid incubated without and with buffer were indicated as–B and +B, respectively. M, marker.

To evaluate the restriction enzyme activity of the six clinical strains isolated in Japan and ATCC700970 strain, cells were recovered from cultures at the exponential phase, washed in PBS, lysed with 1% Triton X-100, mixed with a pT7Blue plasmid and L buffer (Takara Bio, Shiga, Japan), and incubated at 37°C for 1 h. The products were separated using 1% agarose gel electrophoresis (Fig 4). The result indicated that pT7Blue plasmid was cleaved by five of the seven strain lysates, suggesting that restriction enzymes were expressed in the *U*. *parvum* serovar 3 strains. Of these five strains, SN5 was isolated from an endotracheal aspirate of a preterm infant, F26 was isolated from a vaginal swab after an infectious abortion, OMC-P162 was isolated from the human placenta of a preterm delivery at 26 weeks’ gestation, S104 was isolated from a vaginal swab at 8 weeks’ gestation, and S29 was isolated from amniotic fluid at 32 weeks’ gestation. The cleavage patterns from the cell lysates derived from F26, OMC-P162, S104, and S29 were consistent suggesting that these four strains might express the same restriction enzyme.

### Identification of the restriction enzyme protein

**Fig 5:**
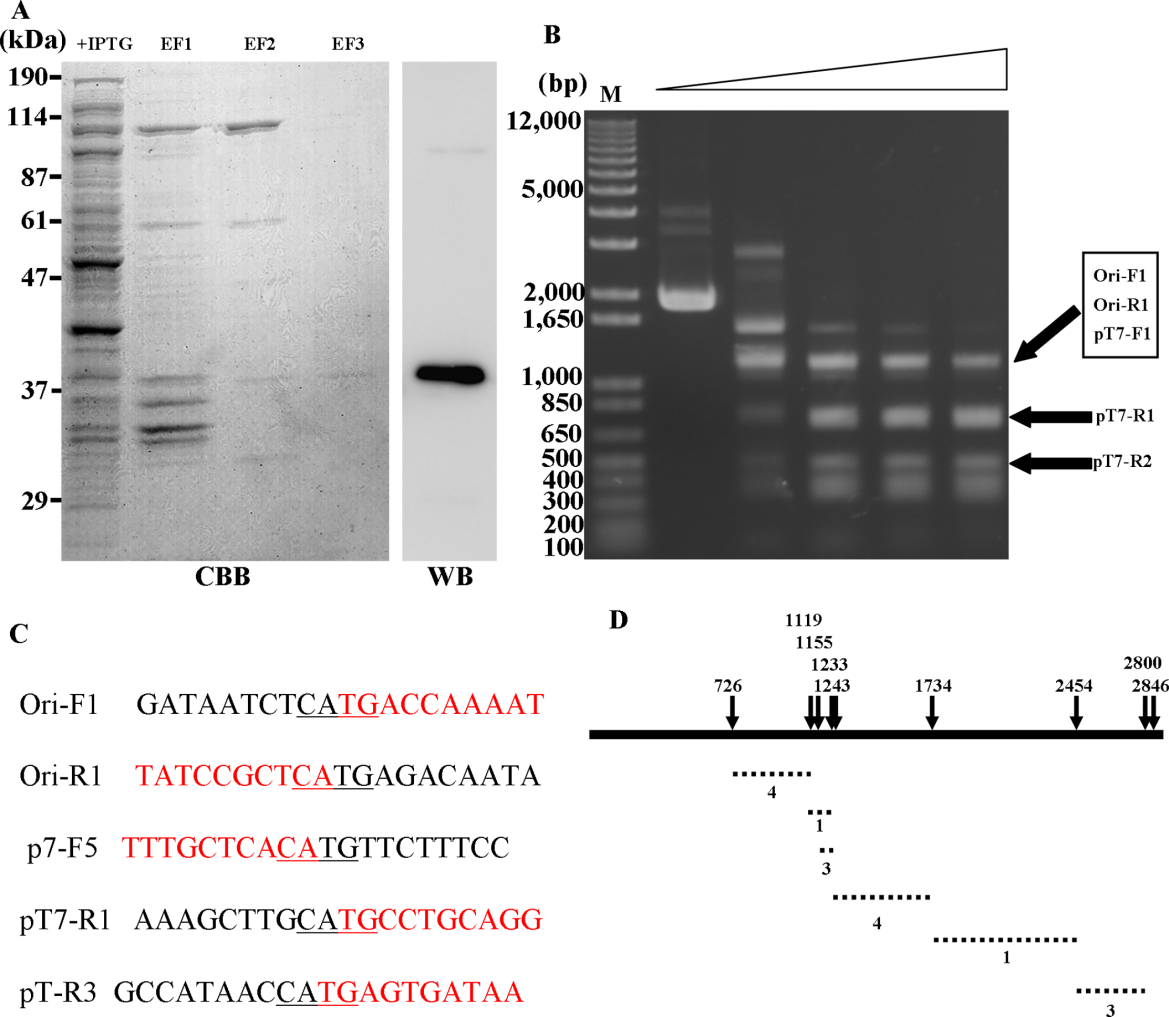
Endonuclease activity of UPV230 protein. (A) SDS-PAGE analysis and western blot of recombinant protein that predicated restriction enzyme. +IPTG: whole cell lysate with IPTG induction, EF1: elution fraction from column filled with cOmplete His-Tag Purification Resin, EF2: elution fraction from column filled with Macro-Prep High S Support, EF3: elution fraction from column filled with heparin, and purified recombinant protein was confirmed by anti-His antibody. (B) Examination of endonuclease activity. pT7Blue plasmid was treated with different concentrations (0, 22.4, 33.6, 44.8 and 89.6 mg/ml) of recombinant UPV230 protein for 1 h, and products were separated by 1% agarose gel electrophoresis. The bands were sequenced by the indicated primers (S1 Table), and the bands used to identify the cleavage site were marked by arrows. (C) Sequence at the end of fragment of Enzyme-digested pT7Blue plasmid. The ends of sequenced DNA fragments in C were shown in red and their adjacent sequences in pT7Blue plasmid were shown in black, and primers used for sequence were indicated at left. Recognition site was underlined. (D) Enzyme–digested pT7Blue plasmid cloned into the *Eco*RV site of pT7Blue plasmid. pT7Blue plasmid was shown as linear solid line. Nine sites of CATG were marked by solid arrows and their A nucleotide positions in pT7Blue plasmid were shown above the arrows. Insertion DNA fragments were shown as dotted lines and obtained sequences numbers were shown.

To confirm that *UPV_*230 coded for a restriction enzyme, we expressed the *UPV_*230 full-length sequence (S1 Fig) in *E*. *coli* as a His-tagged fusion protein. First, UPV230 protein was expressed in *E*. *coli* cells. SDS-PAGE of the cell lysates revealed that UPV230 His-tagged protein was expressed in IPTG-induced cells but not in cells without IPTG induction (S2 Fig). Next, we purified this protein using a Ni-NTA–sepharose-filled columns and heparin–sepharose-filled columns as described in the Materials and Methods. The protein purification was confirmed by the anti-His antibody (Fig 5A). A final amount of 66 μg protein was obtained from 1,000 ml of culture.

Next, the endonuclease activity of the purified protein was examined. The pT7Blue plasmid was treated with the purified protein at a final concentration of 22.4–89.6 μg/ml at 37°C for 1 h and separated by electrophoresis on 1% agarose. The DNA cleavage was observed, and the cleavage patterns were dependent on the protein concentration (Fig 5B). To identify the cleavage site, the individual cleaved bands were cut out and purified from the agarose gel and sequenced. Five sequences whose information can be used to identify the cleavage site were obtained (Fig 5B). Based on the pT7Blue plasmid sequence, we found that the ends of the fragments of UPV230 recombinant protein-digested sequences from 5ʹ to 3ʹwere CA (Fig 5C), suggesting that the enzyme cleavage site could be CATG, with a blunt cut between A and T. To confirm this prediction, pT7Blue plasmid treated by the enzyme was recovered, ligated to the *Eco*RV site of pT7Blue plasmid, and transformed into competent *E*. *coli* JM109 strain. Sixteen randomly selected positive colonies and plasmids were isolated, and the insertion DNA fragments were sequenced. The corresponding positions of these sequences on the pT7Blue plasmid were identified (Fig 5D). Four sequences corresponded to the plasmid position 726–1,119, three sequences to 1,155–1,233, one sequence to 1,233–1,243, four sequences to 1,243–1,734, one sequence to 1,734–2,454, and three sequences to 2,454–2,800. Based on the plasmid sequence information, we determined that recombinant UPV230 protein cleaved the DNA at the same site (CATG), suggesting that this enzyme is a type II restriction enzyme.

### Purification of the *Upa*P162 restriction enzyme from the OMC-P162 strain

**Fig 6:**
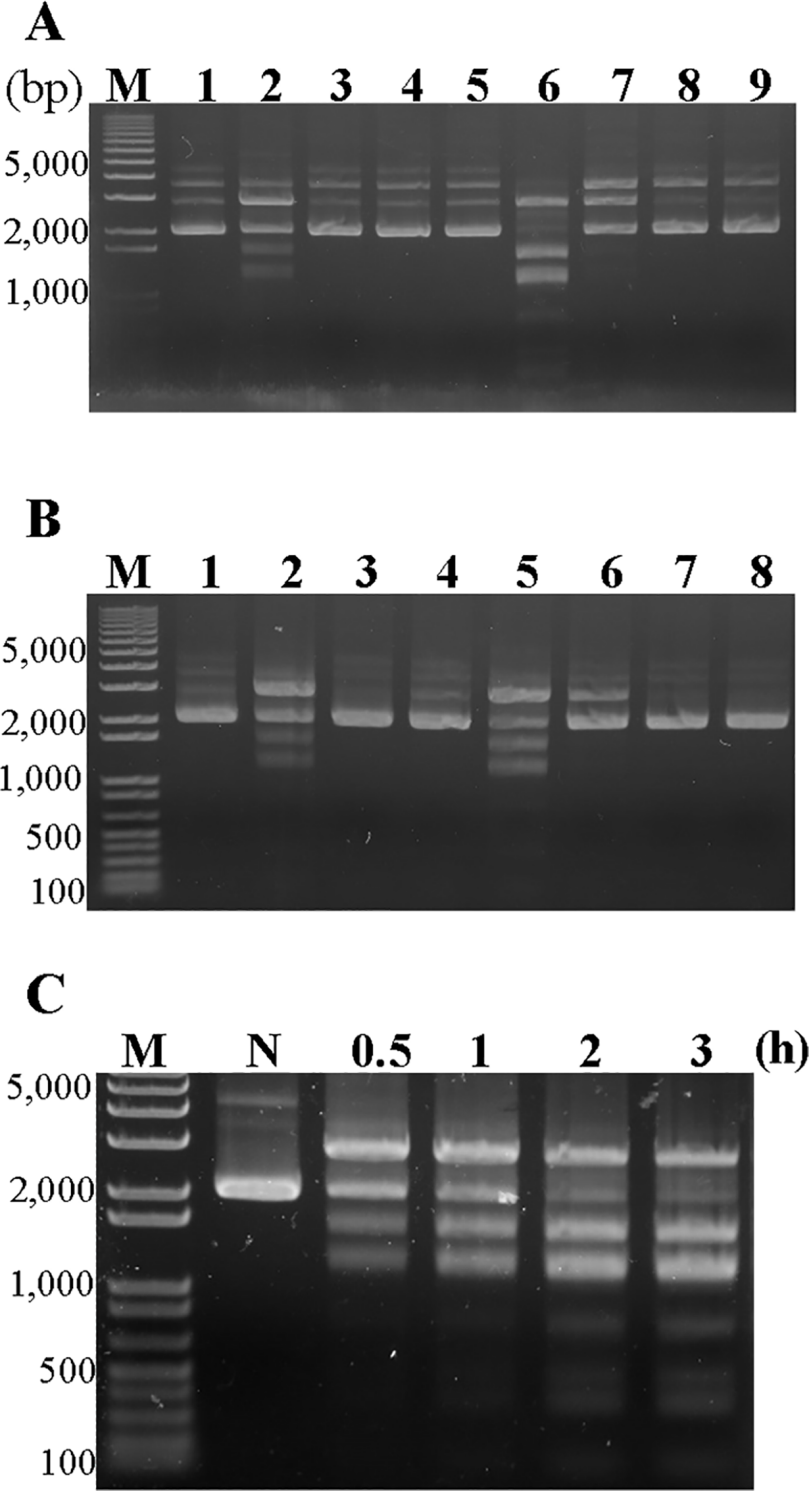
UPV230 naturally expressed in cells. (A) Isolation of enzyme from supernatant by using Macro-Prep High S Support column. The cell lysate was centrifuged, and supernatant was supplied to the equilibrated column, washed by buffer and eluted by gradient concentrations of NaCl. Enzyme-rich fraction was detected by digestion of pT7Blue plasmid in L buffer and products were separated by 1% agarose gel electrophoresis. Lanes contained pT7Blue plasmid as follows: Lane 1, pT7Blue plasmid (negative control). Lanes 2–4, Digestion products of pT7Blue plasmid by using fraction of supernatant, pass through, and washed fraction. Lanes 5–9, Digestion products of pT7Blue plasmid by using elution factions contained 100 mM, 200 mM, 300 mM, 400 mM and 500 mM of NaCl. (B) Isolation of enzyme from enzyme rich fraction from (A) by using Heparin column. Enzyme rich sample was supplied to the equilibrated column, washed by buffer and eluted by gradient concentrations of NaCl. Lanes contained pT7Blue plasmid as follows: lane1, pT7Blue plasmid (negative control). Lanes 2–4, Digestion products of pT7Blue plasmid by using fraction of enzyme rich fraction from (A), passed through and washed fraction. Lanes 5–8 Digestion products of pT7Blue plasmid by using elution factions containing 200 mM, 300 mM, 400 mM and 500 mM of NaCl. M, marker. (C) pT7Blue plasmid was digested with purified enzyme at 37°C for indicated time, and products were separated by 1% agarose gel electrophoresis.

**Fig 7:**
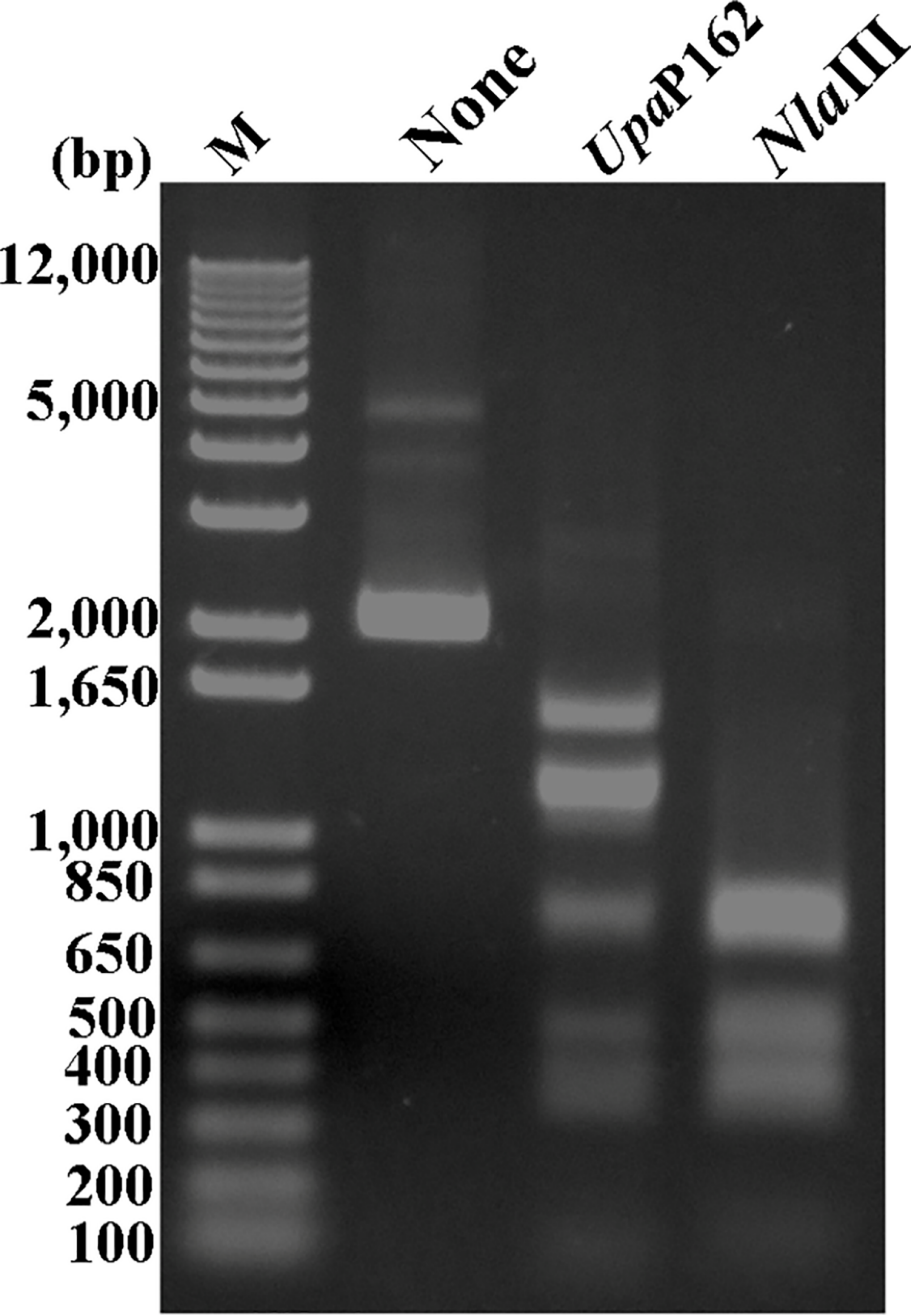
Digestion pattern of pT7Blue by *Upa*P162 and *Nla*III. The pT7Blue plasmid was either untreated or treated for 1 h with either *Upa*P162 or *Nla*III and products were separated by electrophoresis in 1% agarose.

To establish whether the restriction enzyme activity of the UPV230 recombinant protein was identical to that of the cultivated OMC-P162, we purified the restriction enzyme from the OMC-P162 strain. The cells were recovered from a culture at the exponential growth phase, washed with PBS, and lysed with 1% Triton X-100. The supernatant was recovered, diluted 10-fold with PBS, and subjected to ion-exchange chromatography using Macro-Prep High S Support. The restriction enzyme was eluted using gradient concentrations of NaCl and the restriction enzyme activity of each fraction was analyzed (Fig 6A). Next, the restriction enzyme-rich fraction was subjected to affinity chromatography in a heparin–sepharose-filled column, and the restriction enzyme-rich fraction was obtained as described above (Fig 6B). Next, we examined the restriction enzyme activity of the cultivated OMC-P162 strain. The isolated restriction enzyme was mixed with 1 μg pT7Blue plasmid containing L buffer, incubated at 37°C for various times, and the product was separated by 1% agarose gel electrophoresis. The results indicated that the pT7Blue plasmid was digested within the incubation time, and the cleavage pattern was similar between 2 and 3 h of incubation (Fig 6C). Next, we identified the cleavage site of this enzyme. The cleavage pattern of this purified restriction enzyme from OMC-P162 strain was the same as that of the UPV230 recombinant protein. Briefly, enzyme-treated pT7Blue plasmid was recovered, ligated to the *Eco*RV site of pT7Blue plasmid, and the insertion DNA fragments were sequenced. Finally, the cleavage site was identified as CATG, suggesting that the restriction enzyme activity of the UPV230 recombinant protein was identical to that of the cultivated OMC-P162 strain. We designated *UPV_*230 gene encoded restriction enzyme as *Upa*P162. Next, we examined the digestion pattern of the pT7Blue plasmid after treatment with *Upa*P162 or *Nla*III (a neoschizomer of *Upa*P162) at 37°C for 1 h. The cleavage patterns by *Upa*P162 were similar to those of *Nla*III; however, the pT7Blue plasmid was only partially digested by *Upa*P162 (Fig 7).

### The digestion efficiency of *Upa*P162 depends on the sequences around CATG motifs

**Fig 8:**
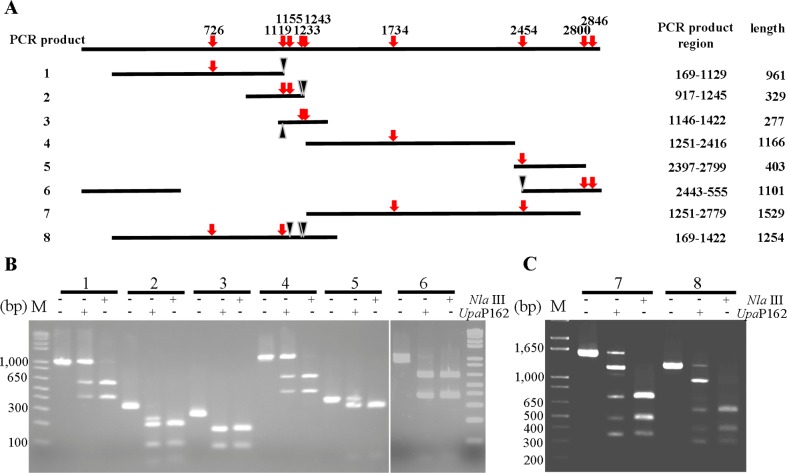
*Upa*P162 activity on CATG motifs. (A) Schematic representation of amplified PCR products 1 to 8. CATG motifs are shown by red arrows and PCR product regions and the sizes, as well as mutation sites (black triangle), are indicated. (B) Digestion of products of 1 to 6 by *UpaP*162 and *Nla*III for 1 h, respectively. (C) Digestion of PCR products 7 and 8 by *Upa*P162 and *Nla*III for 1 h, respectively.

*Upa*P162 partially digested the CATG site within the pT7Blue plasmid (Fig 7). To investigate the mechanism of this incomplete digestion, the bases flanking the CATG motifs in the pT7Blue plasmid were compared using various PCR products. Among the PCR products, products 1, 4 and 5 were designed to contain one CATG motif, and 2, 3 and 6 were designed to contain two (Fig 8A). PCR products 1 to 6 were digested with *Upa*P162, and products 1 and 4, which contain one CATG motif, were only partially digested (Fig 8B). Both of these CATG motifs were flanked by A or T nucleotides. Next, we investigated whether dual CATG motifs in a PCR product influence *Upa*P162 restriction enzyme activity. PCR product 7 was amplified from positions 1,251 to 2,779 of pT7Blue, which includes two CATG motifs localized between 1,734 and 2,454. PCR product 8 was amplified from between 169 and 1,734 of pT7Blue, which includes two CATG motifs localized between 1,233 and 1,243. The CATG sites at 1,155, 1,233 and 1,243 were mutated by overlap PCR (Fig 8C). Neither of these dual CATG motifs enhanced *Upa*P162 activity, suggesting that *UPa*P162 is not a type II E restriction enzyme [22]. TCATGA and ACATGT motifs were not preferentially digested by *Upa*P162 activity.

### Expression of UPV229 and its inhibitory activity against *Upa*P162 restriction enzyme

**Fig 9:**
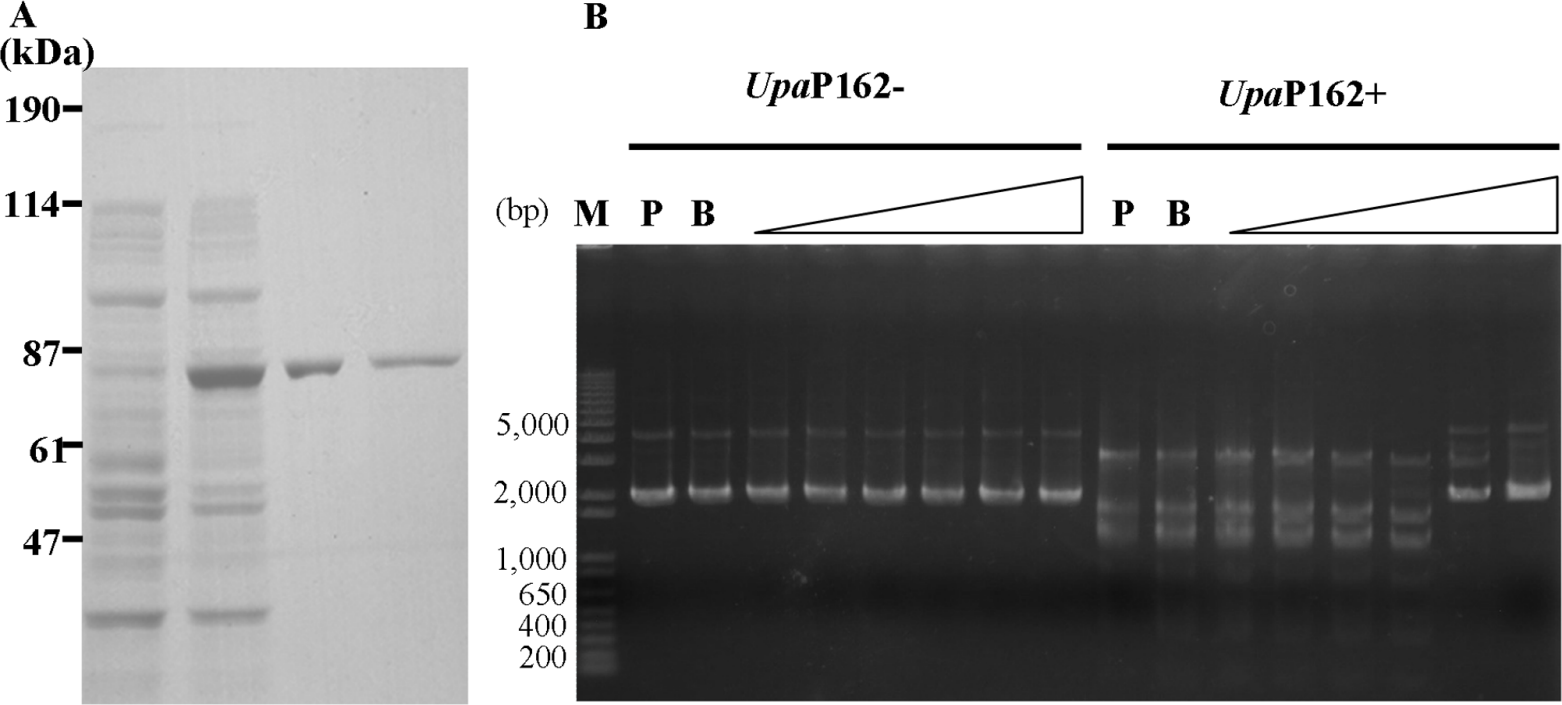
UPV229 protein inhibits the restriction activity of *Upa*P162. (A) SDS-PAGE analysis of cell lysates from *E*. *coli* expressing recombinant UPV229 without and with IPTG induction and purified UPV229 fractions from complete His-Tag Purification Resin and heparin columns (from left to right). (B) Inhibitory restriction enzyme activity of UPV229. Treated pT7Blue plasmid with purified UPV229 protein ranging from 3×10^−7^ to 3×10^−2^ mg/ml was digested by *Upa*P162 for 1 h. P, pT7Blue plasmid was not treated by UPV229 recombinant protein (positive control). B, pT7Blue plasmid was treated with L buffer.

*UPV_*229 was predicted to code for a DNA (cytosine-5)-methyltransferase by gene annotation. To confirm the function of UPV229 protein, we expressed the *UPV_*229 full-length sequence (S1 Fig) in *E*. *coli* as a His-tagged fusion protein. The UPV229 recombinant protein was purified by a Ni-NTA–sepharose-filled columns and a heparin–sepharose-filled column (Fig 9A). Next, we examined the methyltransferase activity of this protein. The pT7Blue plasmid was treated with the recombinant protein UPV229 at a final concentration of 3×10^−6^ to 3×10^−1^ mg/ml, recovered, and digested by *Upa*P162 (Fig 9B). The pT7Blue plasmid that was treated with 0.3 mg/ml of recombinant UPV229 protein completely blocked the *Upa*P162 restriction enzyme activity, suggesting that the UPV229 protein might methylated the DNA. Taken together, the *UPV_*230 and *UPV_*229 played the role of a type II restriction-modification system in the *U*. *parvum* OMC-P162 strain.

### Sensitivity of *Ureaplasma* genomes to digestion by *Upa*P162

**Fig 10:**
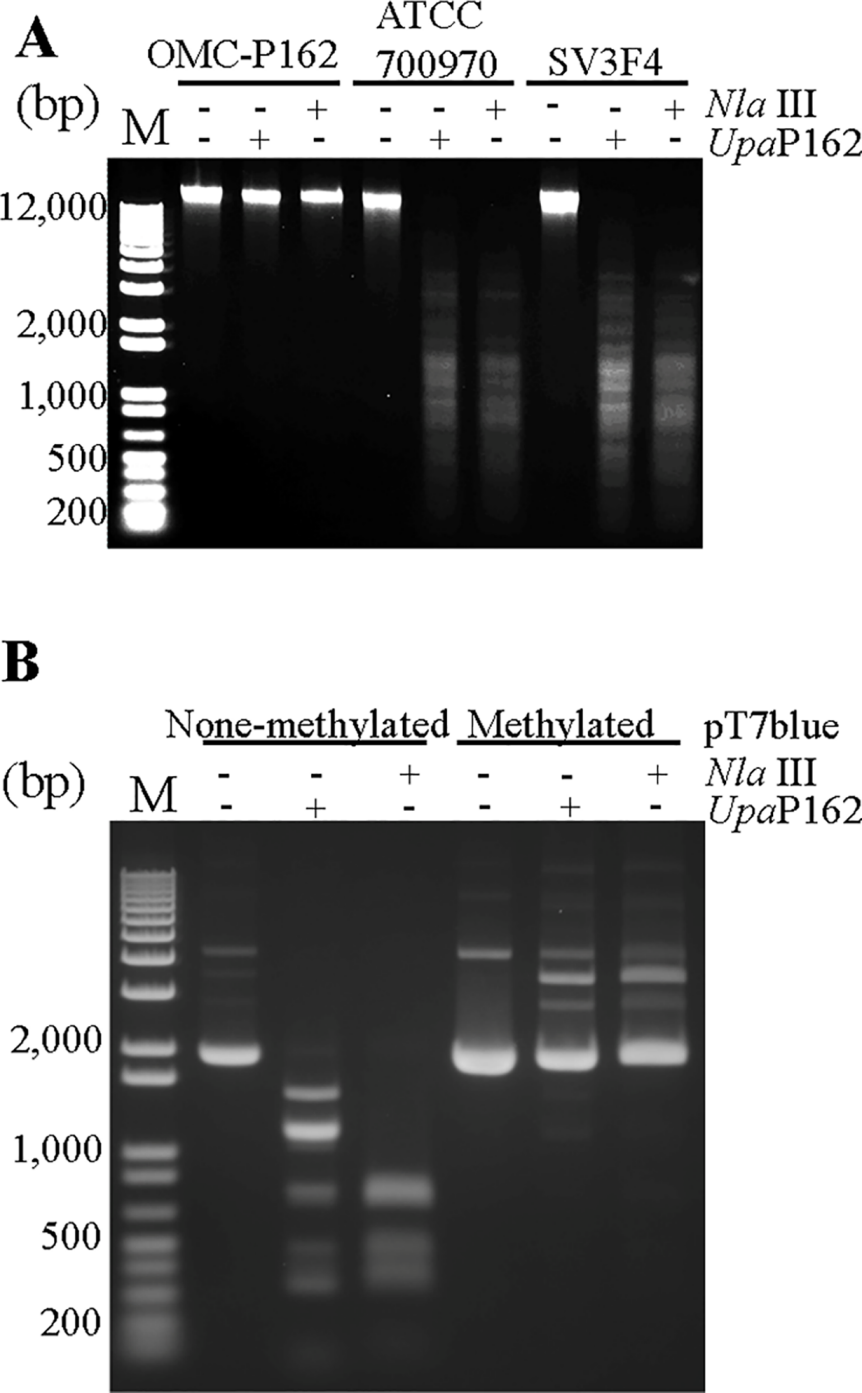
DNA digestion by *Upa*P162 and *Nla*III. **(A)** The indicated *Ureaplasma* genomes were treated with either *Upa*P162 or *Nla*III for 1 h. (B) UPV229-treated and untreated pT7Blue plasmid was digested by either *Upa*P162 or *Nla*III for 1 h.

Given that the UPV229 gene is unique to the OMC-P162 strain and not present in the ATCC700970 and SV3F4 strains (Fig 3A), we isolated the genomes of OMC-P162, ATCC700970 and SV3F4, respectively, and each was digested by *Upa*P162 or *Nla*III. The OMC-P162 genome was resistant to the both of *Upa*P162 and *Nla*III, while the genomes of both ATCC700970 and SV3F4 were susceptible to both of the enzymes (Fig 10A). Additionally, UPV229 was protective from *Nla*III. Indeed, pT7Blue plasmid that had been treated by UPV229 was also completely protected from *Nla*III activity (Fig 10B). *Nla*III is known to be blocked by m6A methylation, suggesting that UPV229 may be an m6A motif-methylation enzyme. Next, we used the *Sph*I restriction enzyme to determine whether m6A is methylated by UPV229. *Sph*I recognizes GCATGC and is inhibited by the presence of m6A. An unmethylated PCR product containing one GCATGC site was digested by both *Upa*P162 and *Sph*I, while prior UPV229 treatment blocked the *Sph*I activity (S3A Fig), suggesting the UPV229 methylated the m6A of GCATGC. Next, we examined whether the OMC-P162 genome is also resistant to *Sph*I by treating OMC-P162 genomic DNA with *Upa*P162, *Sph*I or *Eco*RI, respectively. As expected, the OMC-P162 genome was resistant to both *Upa*P162 and *Sph*I but susceptible to *Eco*RI (S3B Fig).

### *Upa*P162 is a novel restriction enzyme

This is the first report to analyze the restriction-modification (RM) system in *Ureaplasma*. In this study, *UPV_*229 and *UPV_*230 genes act as a type II restriction-modification system in *U*. *parvum* OMC-P162. *UPV_*230 codes for an endonuclease (*Upa*P162) while *UPV_*229 inhibits UPV230. Although a type II restriction enzyme in *Ureaplasma* has been previously reported, the pair genes that code for restriction and modification were not identified [23]. To date, over 3,000 restriction enzymes have been studied in detail [24], and *UPV_*230 did not exhibit any homology to those reported in our Blast search. Furthermore, there was no predicted endonuclease domain for *UPV_*230 using InterPro analysis, suggesting that *Upa*P162 might be a unique restriction enzyme. There are nine CATG sequences in the pT7Blue plasmid, the longest distance between two CATG sequences being 767 bp. However, the longest fragment from the treated pT7Blue plasmid by *Upa*P162 was >1 kb (Fig 4C), suggesting that pT7Blue plasmid did not cut at all CATG sequences. In the present study, *Upa*P162 activity on CATG motifs was examined with PCR products, and we found that TCATGA and ACATGT sequences influence its activity. Characterization of *Ureaplasma* genome has shown that it contains high levels of A and T nucleotides (Fig 1). The GC content of OMC-P162 is only 25.47%. The *Ureaplasma* genome is thought to have reduced its genome size by so-called evolutionary regression, perhaps because an AT rich genome is more unstable than a GC rich structure because of the lesser number of the hydrogen bonds. To protect the AT rich *Ureaplasma* genome from invaders such as phages, it might be advantageous to be able to digest DNAs with contents that are higher in GC than AT. Although the pT7Blue plasmid could not be cut by the cell lysate derived from both SV3F4 and ATCC700970 strains (Fig 1), we cannot rule out the possibility that these two strains could express restriction enzyme activity depending on the buffer used.

### Methylation motifs of UPV229

Homology searching predicted UPV229 to be an m5C methyltransferase; however, as Fig 1B shows, we could only find m4C and m6A methylation in the OMC-P162 genome. This could be explained by m5C methyltransferase being better documented than m4C and m6A. This observation, together with the restriction fragment length polymorphism analysis using *Nla*III and *Sph*I, suggests that the methyltransferase encoded by the *UPV*_229 gene is an m6A methylase. *Sulfolobus acidocaldarius* is an organism that does not have an m5C methylase but does contain an m6A [25]. The methylation status by UPV229 methylase could be unraveled at the molecular level by future mass-spectrometry or NMR analyses.

### Pathogenicity of methylation protein

In this study, we identified different restriction modification activities among *Ureaplasma* spp. (Fig 4). It is known that the DNA methylation proteins play a role in the virulence of bacteria. In *Streptococcus pneumoniae*, *hsds* subunits of Spn556II type -I R-M system causes DNA inversion and excision, and generated variation of Hsd proteins. Hsd proteins alter methylation patterns and the resulting epigenetic switches dictate the regulation of capsule production and modify their virulence [26]. It was observed in a study involving variable surface proteins (Vsa) of *Mycoplasma pulmonis*, a rat pathogen that phylogenetically closely related to *Ureaplasma* spp., play a role in the virulence to escape from host immune system and survive in the host. The restriction and modification enzymes specifically expressed during animal infection are associated with changes in the production of Vsa proteins [27]. Although it is unclear if the deduced methylation protein coded for by *UPV_*229 influences virulence in *Ureaplasma* spp., there are 1,450 CATG motifs in the OMC-P162 genome and 33 of them are localized at the predicted promoter regions (S7 Table). This includes the promoter regions of 50S ribosomal proteins L21, L11, and L10, for 30S ribosomal protein S20 and for a potential virulence protein of type I restriction enzyme S protein. The virulence of UPV229 protein will be the subject of future study.

## Supporting information

S1 Fig Amino acid sequences of UPV229 and UPV230. (TIF)

S2 Fig Expression of recombinant protein. **Related to Fig 4.** C43(DE3) cells harboring pUP162-15 with and without IPTG induction at OD_580_ = 0.5. The cell lysates were subjected to 10% SDS-PAGE, and the expressed proteins were probed with an anti-His antibody.
(TIF)

S3 Fig m6A methylation pattern of UPV229. **Related to Fig 10.** (A) PCR product contained one GCATGC untreated or treated with UPV229 were digested with *Upa*P162 and *Sph*I for 1 h, respectively. (B) The OMC-P162 genome was digested for 1 h by the indicated enzymes.
(TIF)

S1 Table Primers used to identify *Upa*P162-digested sequences and amplify PCR products. Mutant nucleotides are in red bold characters.
(XLSX)

S2 Table Summary modification number related to Fig 1B. (XLSX)

S3 Table Detailed information of genome modifications Fig 1B. (XLSX)

S4 Table Detailed information of m4C modifications of CATG motifs in the genome. (XLSX)

S5 Table Detailed information of m6A modifications of CATG motifs in the genome. (XLSX)

S6 Table Genes and their deduced encoded proteins related to Fig 2. (XLSX)

S7 Table Summary of CATG motifs in promoters. N, not detectable. +, methylated.
(XLSX)
