# Supplementary figures and images for: Type II restriction modification system in Ureaplasma parvum OMC-P162 strain
Source: PLoS One. 2018 Oct 16;13(10):e0205328. doi: 10.1371/journal.pone.0205328 (PMC6191088; doi:10.1371/journal.pone.0205328)

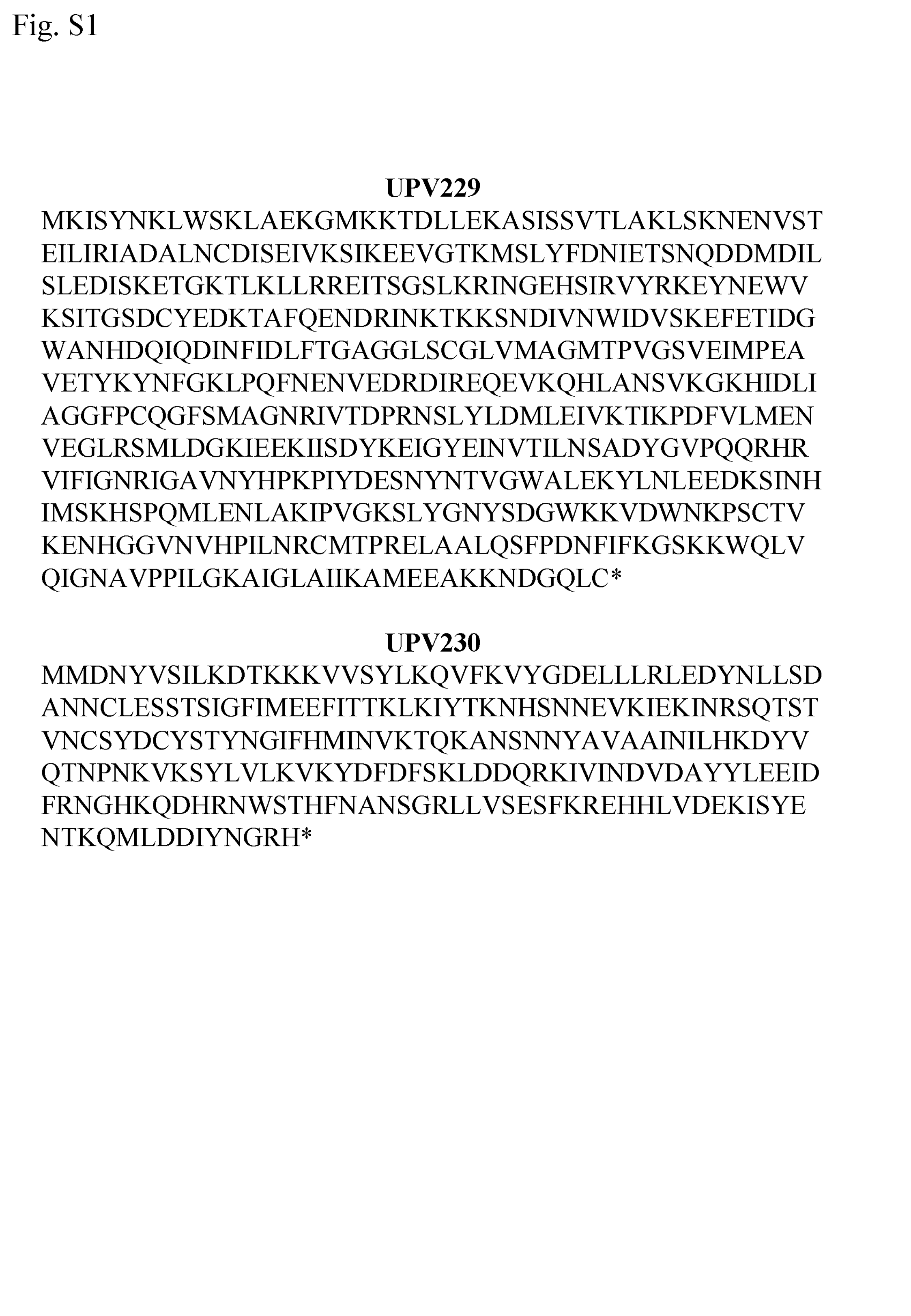

Supplement: S1 Fig — (TIF) [file pone.0205328.s001.TIF]

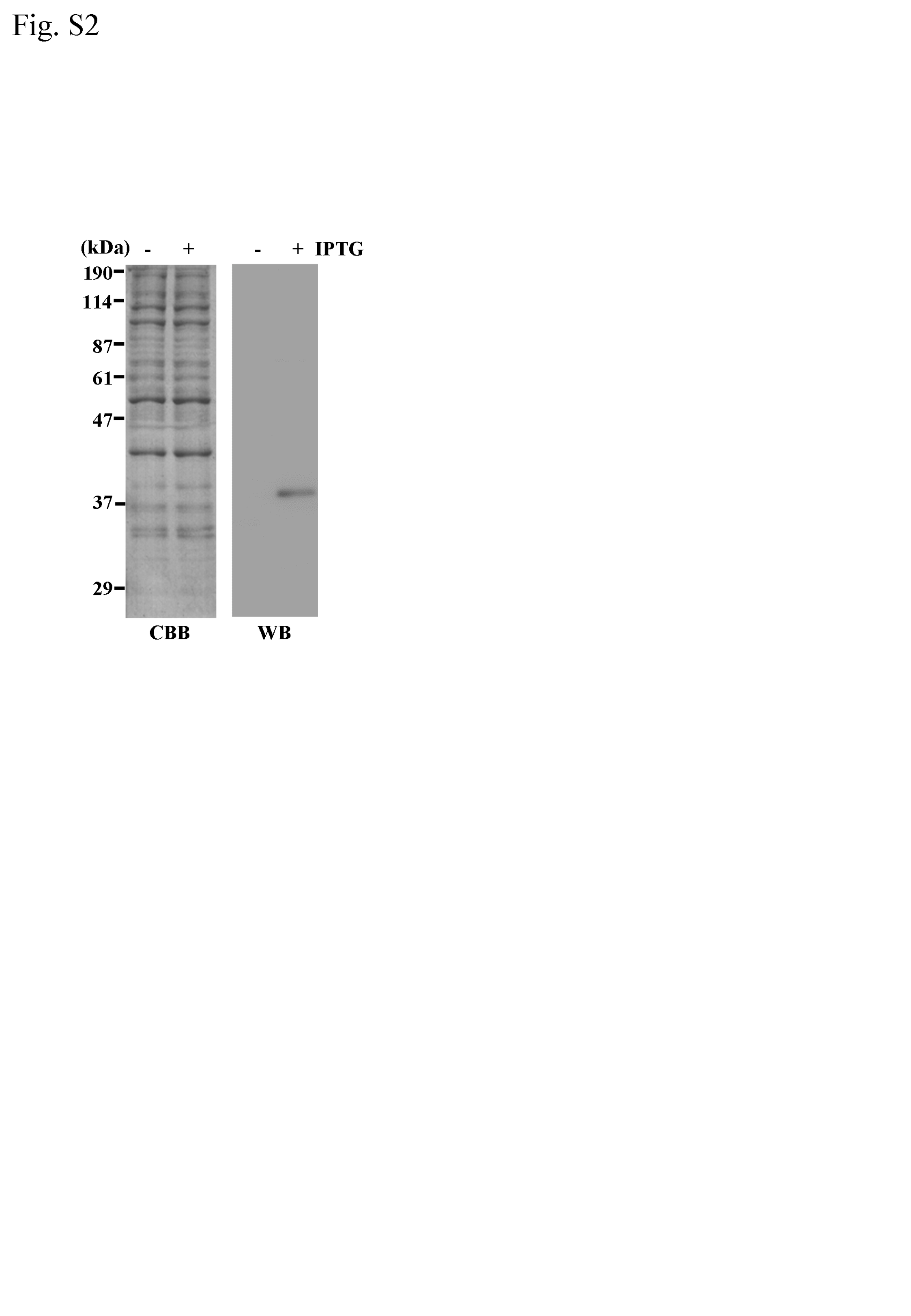

Supplement: S2 Fig — Related to Fig 4. C43(DE3) cells harboring pUP162-15 with and without IPTG induction at OD580 = 0.5. The cell lysates were subjected to 10% SDS-PAGE, and the expressed proteins were probed with an anti-His antibody. (TIF) [file pone.0205328.s002.TIF]

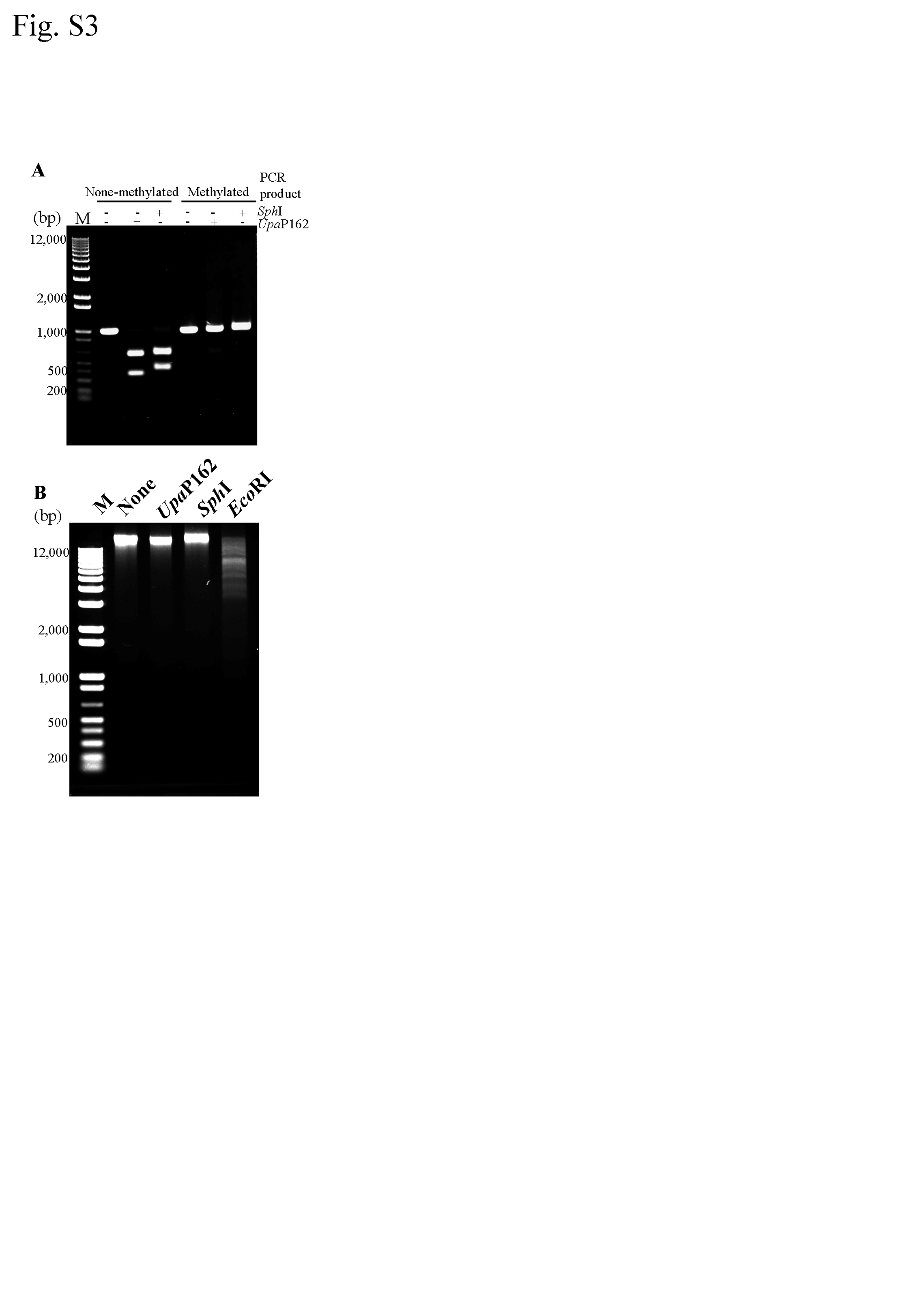

Supplement: S3 Fig — Related to Fig 10. (A) PCR product contained one GCATGC untreated or treated with UPV229 were digested with UpaP162 and SphI for 1 h, respectively. (B) The OMC-P162 genome was digested for 1 h by the indicated enzymes. (TIF) [file pone.0205328.s003.TIF]
